# Prevalence and Factors Associated With Surgical Site Infections in the University Clinics of Traumatology and Urology of the National University Hospital Centre Hubert Koutoukou Maga in Cotonou

**DOI:** 10.3389/fpubh.2021.629351

**Published:** 2021-02-10

**Authors:** Cyriaque Dégbey, Alphonse Kpozehouen, Drissa Coulibaly, Pascal Chigblo, Josué Avakoudjo, Edgard-Marius Ouendo, Aristote Hans-Moevi

**Affiliations:** ^1^Regional Institute of Public Health, University of Abomey-Calavi, Ouidah, Benin; ^2^University Hospital Hygiene Clinic, National University Hospital Centre Hubert Koutoukou Maga, Cotonou, Benin; ^3^University Clinic for Traumatology-Orthopedics and Reconstructive Surgery, Centre National University Hospitalier Hubert Koutoukou Maga, Cotonou, Benin; ^4^University Urology Clinic, National University Hospital Centre Hubert Koutoukou Maga, Cotonou, Benin

**Keywords:** prevalence, factors associated, infection of the surgical site, CNHU-HKM, Benin

## Abstract

**Objectives:** Study the prevalence and factors associated with the occurrence of surgical site infections in University Clinics of Trauma-Orthopedics, Reconstructive Surgery and Urology in a developing country.

**Methods:** This was a retrospective descriptive and analytical study of 384 people operated on during the period of 2019. Logistic regression was used to study the factors associated with surgical site infections. The associations between the dependent variable and the other variables were assessed by the odds ratio (OR) followed by their 95% confidence interval.

**Results:** The prevalence of surgical site infections was 7.81% CI 95% = (5.12–10.51). The factors linked to the surgical site infections in the studied population were the patient&apos;s admission method [OR = 2.74; 95% CI = (1.08–6.95)] and the length of the postoperative stay [OR = 8.75; 95% CI = (2.83–26.98)]. The interview and direct observation identified health care system dysfunctions, medical errors, patient monitoring and financial unavailability as factors that could favor the onset of surgical site infections.

**Conclusion:** Interventions should be focused on the factors identified for the effective management of operated patients.

## Introduction

Health facilities and hospitals are designed to treat and heal the sick. However, it has appeared from the time of the Middle Ages to the present day that they are places where the patient risks an additional infection (1). Nosocomial infections represent a real public health concern with considerable consequences both at the individual level and at the economic level.

Nosocomial or hospital infection is an infection associated with care, absent on admission to hospital. Surgical site infections (SSI) are the leading cause of nosocomial infection among operated patients and the third (14.2%) cause among all hospitalized patients after urinary tract infections (36%) and respiratory infections (12%). Surgical site infections (SSI) are any incisional infection, or any organ or space infection occurring within 30 days of the operation or within 1 year in the event of an implant or implant being placed or prosthetic material (2).

Their surveillance has become, over the past decades, an essential element of any program to control these infections (3). Improving the surveillance and prevention of SSIs in all countries is part of the national program to fight nosocomial infections (4). Prevalence studies remain, despite their limitations, the easiest way to choose within the framework of this surveillance to determine the extent of nosocomial infections, specifically those of operative wounds when resources are limited (1).

In sub-Saharan Africa, economic and social factors are thought to constitute major barriers to the prevention of SSI because of their high incidence (5). In Sub-Saharan Africa, the prevalence of SSI in hospitals ranges from 6.80 to 26%. Significant heterogeneity was observed according to the type of specialty with an incidence of SSIs of 19.1% in general and visceral surgery, 14.8% in orthopedics and 8.6% in obstetric gynecology (6). The prevention of SSI has always been considered as the main axis of the fight against these infections and is today a requirement for healthcare partners (1). In a review of the literature, the incidence of post-cesarean SSI ranges from 3 to 24%; and this, in relation to the often insufficient aseptic conditions in which they are performed (7). Epidemiological studies make it possible to identify risk factors, thus offering the possibility of implementing targeted preventive measures. Each treatment center adapt its prevention strategy according to its own epidemiological data (6). The complications of SSI are feared and despite the progress made in terms of surgical techniques and prevention, their incidence remains high: it is estimated that the overall risk of SSI is 1.2% of patients operated on in France (8). The prevalence of nosocomial infections is significantly higher in developing countries than in developed countries (9).

The objective of this study was to study the prevalence and the factors associated with infections of the surgical site in the University Clinics of Traumatology-Orthopedics and of Reconstructive Surgery and Urology of the National University Hospital Center Hubert Koutoukou Maga (CNHU-HKM) of Cotonou, in order to strengthen ISO prevention measures. The aim is to contribute to the reduction of these postoperative infections and to improve the surgical management of patients.

## Methods

### Study Framework

This study took place at the University Clinics of Traumatology-Orthopedics and Reconstructive Surgery and Urology of the CNHU-HKM. The CNHU-HKM is a public hospital which enjoys legal personality and financial autonomy. The CNHU-HKM offers referral care that cannot be provided in other health centers and is also the main health facility in Benin. To accomplish its mission perfectly, it has a competent medical team and several surgical and medical specialties to which are added the various specialties in diagnostic explorations. It is also the main health facility in the country; it is a center for care, research and training of health workers of all categories. It also leads social actions. Within the National University Hospital Center Hubert Koutoukou MAGA there is a hospital hygiene service created by decree 2006/N °6501/ MS/DC/SGM/CNHU-HKM of July 06th, 2006 whose mission is to participate in the development of the internal hospital hygiene policy under the supervision of the Committee for the Fight against Nosocomial Infections, implement this policy to ensure the promotion of the quality of care and services at the CNHU-HKM in Cotonou. The hospital hygiene service crosses the visceral surgery, urology and trauma services to improve their hygiene quality.

### Type of Study

This was a retrospective descriptive and analytical study covering the year 2019. It took place from April 18th, 2020 to June 12th, 2020.

### Study Population

The study population consisted of primary and secondary targets. The primary target consisted of all the people who had been operated on and hospitalized in the wards of the University Clinics of Traumatology-Orthopedics and of Reconstructive Surgery and Urology in 2019. The secondary target was represented by health personnel (surgeons, supervisors of services, state-certified nurses) university trauma and urology clinics.

### Sampling

The sample consisted of 384 files of operated patients, 02 ward supervisors, 09 surgeons and 21 state-certified nurses. The sampling method was probabilistic with the technique of systematic random choice for operated patients. It was non-probabilistic with the technique of choice for convenience for surgeons and nursing graduates and for reasoned choice for wardens.

### Definition of Variables

The dependent variable was the occurrence of surgical site infections. The criterion for infection was based on purulent discharge from a surgical wound or drain; a discharge obtained from a wound or a drain and whose microbiological culture was positive or not; a wound requiring reopening and/or diagnosis of infection by the surgeon. The independent variables were:

***Factors linked to patients:***age, sex, chronic pathologies (diabetes, renal failure, incontinence, immunosuppression), acute pathologies (burns, multiple trauma) and nutritional status (undernutrition, obesity).

***Care-related factors:***operator qualification, type of surgical intervention, intervention process (duration of the intervention, skin preparation), invasive procedures (bladder probing, endotracheal intubation), associated treatments (corticosteroid therapy, antibiotic therapy, Immunosuppressants).

***Factors related to the knowledge of nursing staff:***knowledge about ISO, attitudes toward ISO, practices toward ISO.

### Collection of Data

Three data collection techniques were used: Observation of the hospital environment, use of documents for patient records, care register and interview with department supervisors, surgeons and state-certified nurses.

### Data Analysis

Data analysis was performed using STATA software version 11. We first described the main study variables using frequency for qualitative variables and central tendency parameters (Mean, median) and dispersion (standard deviation, interquartile range) for the quantitative variables. Then, we performed the bivariate analysis through logistic regression between the occurrence of surgical site infections and each of the independent variables at the 20% level. Finally, the variables which had a *p*-value of < 20% in bivariate analysis were introduced into a stepwise descending multivariate regression model. Variables with a *p*-value ≥ 5% were gradually eliminated until the final model was obtained, which only contained variables whose *p*-value was < 5%. The associations between the dependent variable and the independent variables were assessed by the odds ratio (OR) followed by their 95% confidence interval 95% CI. The final model was adequate if the *p*-value is >5% threshold.

#### Ethical Considerations

Before our study was carried out, a formal authorization request was sent to the administrative authorities of the hospital and to the various heads of departments who gave their consent. The objectives of the study were presented to heads of departments and their staff. Free and verbal consent has been obtained. The information collected in each of the files made available to us was treated with anonymity and strict confidentiality. This is why the count sheets made no mention of the identity of the interviewee.

## Results

### Sociodemographic Characteristics and Clinical Data of the Operated

The average age of the operated was 49 years old with a minimum of 3 years and a maximum of 89 years. Of the total, 53.13% of those operated on were hospitalized at the University Clinic for Traumatology-Orthopedics and Reconstructive Surgery (CUTO-CR). The predominant sex was male with a male/female sex ratio of 4.26. The most representative mode of admission was those who came from themselves, i.e., 84.64%. Of the 384 people operated on, 368 people had undergone the procedure on a scheduled basis. Of those operated on, more than half had no chronic pathology in addition to the surgical disease and 80.63% had ongoing antibiotic therapy. Only 9.64% had an acute pathology associated with the surgical disease (Table 1).

**Table 1:** Sociodemographic characteristics and clinical data of those operated on in the university trauma and urology clinics at the CNHU-HKM in Cotonou in 2019.

**Variables**	***n***	**%**
**Sex**		
Male	311	80.99
Female	73	19.01
**Patient admission method**		
Self presentation	325	84.64
Transfer by another center	59	15.37
**Nature of intervention**		
Urgent	15	3.92
Programmed	368	96.08
**Service**		
University Urology Clinic	180	46.88
University Traumatology Orthopedy Clinic	204	53.13
**Pathology acute associated**		
Polytraumatism	37	9.64
None	347	90.36
**Under treatment**		
Immunosuppressants	8	2.09
Corticoids	13	3.40
Antibiotiques	308	80.63
None	53	13.88
**Associated chronic pathology**		
Renal failure	4	1.04
Diabetes	15	3.91
Urinary incontinence	1	0.26
Heart disease	55	14.32
None	297	77.34
Other	12	3.13

### Features Related to the Surgical Procedure

The duration of surgery was >2 h in 85.18% of patients. More than half of the patients (56.92%) had a postoperative stay of 1 week. The dressing was changed every day in 87.87% of the operated patients. Shaving of the operating field was performed in 50.26% of cases. Urinary catheterization was performed in 208 operated patients, 125 received an implant and 45 a drain. The patient discharge method was exeat in 98.69% of cases. Almost all of the operated patients did not receive intraoperative antibiotic therapy, i.e., 97.75% (Table 2).

**Table 2:** Characteristics related to the surgical act in those operated on in the University Clinics of Traumatology and Urology at the CNHU-HKM of Cotonou in 2019.

**Variables**	***n***	**%**
**Duration of intervention**		
Between 1 and 2 h	55	14.82
≥ 2 h	316	85.18
**Postoperative length of stay**		
1 week	185	56.92
2 weeks	71	21.85
3 weeks	29	8.92
4 weeks	17	5.23
≥5 weeks	23	7.08
**Preoperative length of stay**		
1 day	48	14.50
>1 day	283	85.50
**Bandage rhythm**		
Each day	326	87.87
1 day of 2	37	9.97
1 day of 4	8	2.16
**Shaving of the operating site**		
No	190	49.74
Yes	192	50.26
**Placement of a urinary catheter**		
Yes	208	54.17
No	176	45.83
**Placement of an implant or prosthesis**		
No	258	67.36
Yes	125	32.64
**Installation of a drain**		
No	338	88.25
Yes	45	11.75
**Patient discharge mode**		
Dead	5	1.31
Exeat	377	98.69
**Intraoperative antibiotic therapy**		
No	347	97.75
Yes	8	2.25

### Characteristics Linked to the Infection of the Surgical Site

The infection was accompanied by fluid discharge in 69.57% of cases. The organs most affected by infection were the skin and subcutaneous tissue above aponeurosis in 87.50% of infected cases. The local signs of infection most frequently mentioned by those operated on were pain and swelling.

### Prevalence of Surgical Site Infections

Among the 384 operated, 30 developed an infection of the surgical site (7.81%), 95% CI = (5.12–10.51).

### Relationship Between the Infection of the Surgical Site, the Socio-Demographic Characteristics and the Clinical Data of the Operated

Factors related to surgical site infections were presented in Table 3. Factors such as patient age, patient admission method and department were significantly associated with surgical site infection. Patients who were over 60 years of age were at greater risk [OR = 19.17; 95% CI = (10.25–35.85)] of having an infection of the surgical site than those who were under 30 years of age when taking into account the other variables. Patients transferred from another centers were at greater risk [OR = 3.11; 95% CI = (1.37–7.04)] of having a surgical site infection than those who came from themselves. Surgical patients hospitalized in orthopedic trauma were at greater risk [OR = 2.59; 95% CI = (1.12–5.99)] of having an infection of the surgical site than those operated on hospitalized in urology.

**Table 3:** Relationship between the infection of the surgical site, the socio-demographic characteristics and the clinical data of those operated on in the University Clinics of Traumatology and Urology at the National University Hospital Center Hubert Koutoukou Maga in Cotonou in 2019.

**Variables**	**OR**	**Confidence interval**	***p*-Value**
**Age**			
<30 years	1	–	
30–60 years	5.48	(2.40–12.52)	0.000^*^
>60 years	19.17	(10.25–35.85)	0.000^*^
**Sex**			
Male	1	–	
Female	1.94	(0.84–4.43)	0.115
**Profession**			
Officials	1		
Housewife	4.81	(0.85–27.09)	0.074
Farmers	2.52	(0.22–27.72)	0.449
Students	2.40	(0.45–12.87)	0.304
Trader	1.02	(0.39–5.16)	0.585
**Patient admission method**			
Self presentation	1	–	
Transfer by another center	3.11	(1.37–7.04)	0.006^*^
**Under treatment**			
Antibiotics	1	–	
Corticoids	0.70	(0.08–5.89)	0.743
None	0.19	(0.01–3.49)	0.226
**Service**			
University Urology Clinic	1	–	
University Traumatology Orthopedy Clinic	2.59	(1.12–5.99)	0.025^*^
**Pathology acute associated**			
Polytraumatism	1	–	
None	0.04	(0.01–1.09)	0.944

* *Significant*.

### Relationship Between the Infection of the Surgical Site and the Characteristics Related to the Care of the Operated on

Table 4 shows the relationship between the infection of the surgical site and the characteristics of the care of the operated on. Factors such as patient discharge method, urinary catheter placement, length of preoperative stay, rate of dressing, and duration of surgery were not significantly associated with surgical site infection. Patients who did not shave the surgical site before surgery were 2.52 times more likely to have a surgical site infection than those who had shaved [OR = 2.52; 95% CI = (1.12–5.67)]. In our study, shaving was done in the operating room on the day of the operation after the patient had settled in with a BIC razor. Urgently operated patients were 1.20 times more likely to have a surgical site infection than those who were scheduled [OR = 1.20; 95% CI = (1.01–4.10)]. Patients who had a stay of at least 3 weeks were at high risk of developing an infection. Patients who had a postoperative length of stay of more than 5 weeks were 8.55 times more likely to have an infection of the surgical site than those who had a duration of 1 week when taking into account the other variables [OR = 8.55; 95% CI = (2.81–26.02)].

**Table 4:** Relationship between the infection of the surgical site and the characteristics linked to the care of the operated on in the university trauma and urology clinics at the National University Hospital Center Hubert Koutoukou Maga in Cotonou in 2019.

**Variables**	**OR**	**Confidence interval**	***P*-Value**
**Exit mode**			
Healed	1	–	
Exeated	0.12	(0.01–1.15)	0.512
**Shaving of the operating site**			
Shaving of the operating site	1	–	
No shaving of the operating site	2.52	(1.12–5.67)	0.025^*^
**Nature of intervention**			
Programmed	1	–	
Urgent	1.20	(1.01–4.10)	0.000^*^
**Urinary catheter**			
No urinary catheter	1	–	
Presence of urinary catheter	0.83	(0.41–1.67)	0.601
**Preoperative length of stay**			
1 day	1	–	
>1 day	1.04	(0.01–1.10)	0.656
**Postoperative length of stay**			
1 week	1	–	
2 weeks	0.86	(0.22–3.28)	0.829
3 weeks	4.07	(1.26–13.17)	0.019^*^
4 weeks	6.01	(1.63–22.20)	0.007^*^
≥5 weeks	8.55	(2.81–26.02)	0.000^*^
**Bandage rhythm**			
Each day	1	–	
1 day of 2	1.04	(0.03–2.52)	0.232
1 day of 4	1.09	(0.05–5.85)	0.661
**Duration of intervention**			
Between 1 and 2 h	1	–	
≥2 h	1.03	(0.01–2.03)	0.190

* *Significant*.

The variables retained in the model were presented in Table 5. By introducing the independent variables significantly associated with the occurrence of SSI, only the patients transferred by another centers [OR = 2.74; CI 95% = (1.08–6.95)] and the length of postoperative stay of 5 weeks [OR = 8.75; 95% CI = (2.83–26.98)] were independent predictors of the occurrence of SSI. Indeed, the analysis of the results of the multivariate analysis reveals that the patients transferred by another center were 2.74 times more at risk of having an infection of the operating site than those who had come on their own by adjusting to the other variables of the model. Adjusted for the other variables in the model, patients who had a postoperative length of stay of 5 weeks and more were 8.75 times more likely to have a surgical site infection than those who had a 1 week length of stay while taking into account the other variables.

**Table 5:** Variables associated with infection of the surgical site in the National University Hospital Center Hubert Koutoukou Maga trauma-orthopedic and urology university clinics in Cotonou in 2019 in the final model.

**Variables**	**OR**	**Confidence interval**	***P*-value**
**Patient admission method**			
Self presentation	1	–	
Transfer by another center	2.74	(1.08–6.95)	0.034^*^
**Duration of postoperative stay**			
1 week	1	–	
2 weeks	0.79	(0.20–3.06)	0.743
3 weeks	3.55	(1.07–11.75)	0.037^*^
4 weeks	4.70	(1.22–18.11)	0.024^*^
≥5 weeks	8.75	(2.83–26.98)	0.000^*^

* *Significant*.

### Assessment of the Staff's Level of Knowledge of Surgical Site Infections

During the interviews with the resource persons of the Traumatology, Orthopedics and Urology Clinics of the CNHU-HKM in Cotonou, our findings were as follows:

▪ The nursing staff of the University Clinics of Traumatology, Orthopedics and Urology did not benefit from training on nosocomial infections in general and on those of the operating site in particular. Despite the existence of the transversal hospital hygiene service in the other services which ensures the strict application of hygienic measures, ISOs continue to be recorded.
▪ The factors and situations incriminated in the occurrence of surgical site infections depend on the patients, the staff and the organization of the services.
▪ The stages of decontamination, cleaning and sterilization of medico-technical equipment were respected.
▪ Plastic trash cans without lids are a reservoir of germs that are opened in the dressing room.

## Discussion

### Prevalence of Surgical Site Infection

The prevalence of infection of the surgical site in the University Clinics of Traumatology-Orthopedics and Urology at the CHU-HKM of Cotonou in 2019 was 7.81%. Haidara D in his study on surgical wound infections at the Ouidah zone hospital in 2008 reported a prevalence of 22.8% (1), which is higher than that of our study. This difference could be explained by the fact that his study was carried out at the lowest level of the health pyramid where the technical platform is failing. A study carried out in 2018 at the CNHU-HKM in the University Trauma Clinic reported a prevalence of 9.59% which was higher than that obtained in our study (10). This could be explained by the efforts made in infection prevention and control at the hospital level. In Tunisia, Latifa et al. reported a prevalence of 19.1% of SSIs in general and visceral surgery and 14.8% in Orthopedics (11). These prevalences are above that of our study, the difference could be explained by their sample size which was relatively larger than that of our study. In Benin, a study carried out at the Ouémé and Plateau Departmental Hospital Center reported a prevalence of surgical site infections of 33.8% (12). In Tunisia, Houet et al. reported a prevalence of infection of the operative site in digestive surgery of 3.53% (11), which is far below that obtained in our study. The prevalence of SSIs obtained in the present document is far below that found at CHU HASSAN II in Fez, which is 46% (3). The prevalence of surgical site infections in our study was higher than that found by Hodonou et al. at the Borgou Departmental Hospital Center in Parakou (Benin) in 2016 (13).

### Patient Risk Factors

In our study, patients who were ≥60 years of age were at greater risk of having a surgical site infection than those aged 30–60. This result was in agreement with the data reported in the literature (1). The results of our study revealed that gender was not statistically associated with surgical site infection. This result corroborates that of Birintanya who had this association during her study in 2002 (12). This could be explained by the fact that the majority of patients operated on in the Traumatology-Orthopedics and Reconstructive Surgery Clinics were exclusively male. The significant association of surgical site infection with the inpatient department (CUTO-CR) in our study would be due to the large number of men in our sample. The current treatment and the acute pathology in the operated on were not associated (*p* = 0.94) in our study, which could be explained by an incomplete preoperative assessment or sometimes non-existent for the search for other causes before surgery. In our study, patients who had no ongoing treatment were 0.19 times more likely to have a surgical site infection than those who had antibiotics.

### Risk Factors Related to the Surgery

Preoperative shaving also influences the operative risk. In our study, patients who had not had their surgical site shaved prior to surgery were at greater risk of developing a surgical site infection than those who had shaved. In our study, shaving was done in the operating room on the day of the operation after the patient had settled in with a BIC razor. This was in accordance with the results of other studies (1, 14). Several studies have shown an increased risk when hair is removed with a hand razor compared to an electric razor or hair removal. In addition, when a hand razor was used, the risk was two-fold when the shaving was done in the 24 h before the operation compared to immediately before the operation. Infection of the operative site prolongs the length of postoperative stay. The longer the postoperative stay, the greater the risk of infection of the site. Thus, in our study, we found that patients who had a postoperative length of stay of 5 weeks and more were more at risk of having an infection of the surgical site than those who had a duration of 1 week, as reported by several authors in their studies (1, 10, 15). The risk of surgical site infection appears to be particularly increased for operations lasting more than 2 h. In our study, the risk of an operation lasting longer than 2 h was 1.03. Several factors are mentioned to explain this increased risk: increase in wound contamination, intensification of surgical trauma, increase in the number of sutures, increase in blood loss, decrease in the effect of prophylactic antibiotics. This result is consistent with that reported by other studies (1, 16). A prolonged preoperative stay would increase the risk of infection apart from the presence of other risk factors. In our study, we did not find a significant association between length of preoperative stay and surgical site infection. The same observation was made by Debarge et al. (17). However, in some studies, this association has been mentioned as significant and in the literature (1, 13, 18, 19). This difference could be explained by the difficulty associated with determining the date of admission before the operative act in our study.

### Predictive Model of Surgical Site Infection

The factors that we have identified are clearly found as risk factors in the literature such as the length of postoperative stay (1, 4, 13, 15, 16) and the mode of admission of the patient (1, 4). Interviews carried out with the staff of the University clinics of Traumatology, Orthopedics and Urology of the CNHU-HKM, revealed that there is a need for continuous training of staff on nosocomial infections in order to be able to strengthen their knowledge of the risk infectious. Despite the capacity building of staff by the University Hospital Hygiene Clinic and the Committee for the Fight against Nosocomial Infections (CLIN), much remains to be done in raising the awareness of nursing staff on the prevention and control of infections. It would have been interesting to know the attitudes, knowledge and practices of patients and caregivers, which would better reflect the environment in which the operated person operates. Other studies (1, 19) had suggested that hand hygiene between two dressings and the wearing of sterile gloves would significantly reduce the frequency of nosocomial infections. This is not systematic in most of our health facilities and hospitals.

## Conclusion

Nowadays, surgical site infections continue to be a major public health problem in Benin. Among the 384 operated, 30 developed an infection of the surgical site (7.81%). The factors identified were focused mainly on the current treatment, the type of wound, the associated chronic pathology, the mode of admission, the profession, the department, the mode of discharge of the patient, the shaving of the operating site and the rhythm of the bandage. We also underlined the lack of knowledge of surgical site infections by the staff and the need for training in this health phenomenon. These results show the persistence of patient and care factors in the occurrence of surgical site infections. Hygiene measures were insufficient, which would increase the risk of the occurrence of SSIs. It is urgent to invest in the prevention and adequate management of these factors for a significant improvement in health.

## Data Availability Statement

The raw data supporting the conclusions of this article will be made available by the authors, without undue reservation.

## Ethics Statement

Ethical review and approval was not required for the study on human participants in accordance with the local legislation and institutional requirements. Written informed consent for participation was not required for this study in accordance with the national legislation and the institutional requirements.

## Author Contributions

CD is the principal investigator and participated in the planning and conducts of the study. DC performed the data entry and analysis. PC, AK, JA, E-MO, and AH-M participated in the planning of the study and contributed to the writing process. All authors contributed to the article and approved the submitted version.

## Conflict of Interest

The authors declare that the research was conducted in the absence of any commercial or financial relationships that could be construed as a potential conflict of interest.
